# The FMRF-NH_2_ gated sodium channel of *Biomphalaria glabrata*: Localization and expression following infection by *Schistosoma mansoni*

**DOI:** 10.1371/journal.pntd.0011249

**Published:** 2023-06-23

**Authors:** Laura C. Vicente-Rodríguez, Amanda C. Torres-Arroyo, Anthony Hernández-Vázquez, Mariela Rosa-Casillas, Dina P. Bracho-Rincón, Paola Méndez de Jesús, Martine L. Behra, Mohamed R. Habib, Xiao-Nong Zhou, Joshua J. C. Rosenthal, Mark W. Miller

**Affiliations:** 1 Institute of Neurobiology University of Puerto Rico, Medical Sciences Campus San Juan, Puerto Rico; 2 Department of Anatomy & Neurobiology University of Puerto Rico, Medical Sciences Campus San Juan, Puerto Rico; 3 Medical Malacology Department, Theodor Bilharz Research Institute Giza, Egypt; 4 National Institute of Parasitic Diseases Chinese Center for Disease Control and Prevention Shanghai, People’s Republic of China; 5 Eugene Bell Center Marine Biological Laboratory Woods Hole, Massachusetts, United States of America; University of Liverpool, UNITED KINGDOM

## Abstract

The neglected tropical disease schistosomiasis impacts over 700 million people globally. *Schistosoma mansoni*, the trematode parasite that causes the most common type of schistosomiasis, requires planorbid pond snails of the genus *Biomphalaria* to support its larval development and transformation to the cercarial form that can infect humans. A greater understanding of neural signaling systems that are specific to the *Biomphalaria* intermediate host could lead to novel strategies for parasite or snail control. This study examined a *Biomphalaria glabrata* neural channel that is gated by the neuropeptide FMRF-NH_2_. The *Biomphalaria glabrata* FMRF-NH_2_ gated sodium channel (*Bgl-*FaNaC) amino acid sequence was highly conserved with FaNaCs found in related gastropods, especially the planorbid *Planorbella trivolvis* (91% sequence identity). In common with the *P*. *trivolvis* FaNaC, the *B*. *glabrata* channel exhibited a low affinity (EC_50_: 3 x 10^−4^ M) and high specificity for the FMRF-NH_2_ agonist. Its expression in the central nervous system, detected with immunohistochemistry and *in situ* hybridization, was widespread, with the protein localized mainly to neuronal fibers and the mRNA confined to cell bodies. Colocalization of the *Bgl-*FaNaC message with its FMRF-NH_2_ agonist precursor occurred in some neurons associated with male mating behavior. At the mRNA level, *Bgl-*FaNaC expression was decreased at 20 and 35 days post infection (dpi) by *S*. *mansoni*. Increased expression of the transcript encoding the FMRF-NH_2_ agonist at 35 dpi was proposed to reflect a compensatory response to decreased receptor levels. Altered FMRF-NH_2_ signaling could be vital for parasite proliferation in its intermediate host and may therefore present innovative opportunities for snail control.

## Author summary

Schistosomiasis, or ‘snail fever’, is caused by parasitic flatworms known as schistosomes. Presently, there is no effective vaccine for this major disease of poverty. The trematode worm that causes the most widespread form of schistosomiasis, *Schistosoma mansoni*, depends upon a specific pond snail, *Biomphalaria*, to support a part of its life cycle. Within the snail, larval schistosomes multiply and transform into a form that can penetrate human skin. The disease could therefore be mitigated with effective strategies to control this intermediate host snail. This study forms part of a research program designed to identify specific targets in the *Biomphalaria* nervous system that could disrupt the schistosome life cycle. The present investigation explored the properties of an unusual neuropeptide receptor, the FMRF-NH_2_ activated sodium channel (FaNaC). This class of receptors may provide opportunities for highly specific pharmacological or genetic snail control interventions that have minimal impact on other animal groups.

## Introduction

Pond snails of the genus *Biomphalaria* (Mollusca: Gastropoda: Heterobranchia; Planorbidae) serve as intermediate hosts for *Schistosoma mansoni*, the causative agent for the most widespread form of intestinal schistosomiasis [1,2]. Within their snail hosts, larval trematodes multiply and transform into the cercarial form that can infect humans [3,4]. Strategies for elimination of schistosomiasis include improved sanitation, large-scale preventive chemotherapy, and snail control [5,6,7].

Neuropeptide signaling systems are promising molecular targets for pesticide and parasiticide drug development [8,9,10]. In contrast to the classical neurotransmitter systems that are presently common targets for pest control, some neuropeptides and their receptors are limited to specific invertebrate clades, reducing concerns of widespread toxicity [10,11,12]. The FMRF-NH_2_ family of neuropeptides holds potential for drug development due to its pervasive role in the behavior and neuromuscular physiology of major arthropod and helminth parasites and pests [13,14,15]. FMRF-NH_2_ was initially purified from a bivalve mollusc (sunray venus clam, *Macrocallista nimbosa*; [16]) and has been intensively studied in several gastropod species [17,18,19]. To date, however, the potential utility of this peptide signaling system for snail control interventions has not been explored.

In common with most neuropeptides, the gastropod FMRF-NH_2_-related peptides (FaRPs) activate G-protein coupled receptors (GPCRs) that regulate ion channels via second messenger systems [20,21,22,23]. In addition, FMRF-NH_2_ directly activates a member of the degenerin / epithelial sodium channel (DEG/ENaC) superfamily of channels that do not require second messenger signaling [24,25,26,27]. One such channel, termed the FMRF-NH_2_-activated amiloride-sensitive sodium channel-like protein, was recently identified in the genome of *Biomphalaria glabrata* ([28]; GenBank Accession number XP_013063507).

Ionotropic DEG/ENaC channels are expressed in numerous cell types and tissues and are gated by varied stimuli, including mechanical forces and protons [29,30,31]. In the nervous system, members of this channel family participate in a range of functions, including mechanotransduction, nociception, and synaptic plasticity [32,33,34]. Involvement of the FMRF-NH_2_ activated sodium channel (FaNaC) in the neural circuits that regulate gastropod physiology and behavior has not been established.

Recently, we examined the precursor organization of the *B*. *glabrata* FMRF-NH_2_-related neuropeptides and localized their expression in the CNS [35]. As our understanding of this neuropeptide system will be broadened by defining its complementary receptors, the present study characterized the *B*. *glabrata* FMRF-NH_2_-activated sodium channel, localized its expression in the CNS, and explored whether its expression may be modified following exposure to *Schistosoma mansoni*.

## Materials and methods

### Ethics statement

All protocols were approved by the Institutional Animal Care and Use Committee (IACUC) of the University of Puerto Rico Medical Sciences Campus (Snails: Protocol #3220110; *Xenopus*: Protocol #9470110). All animal care and experimental procedures followed guidelines and regulations specified in the *Guide for the Care and Use of Laboratory Animals* (National Research Council) [36].

### Specimens

Histological protocols were performed on *B*. *glabrata* snails bred in the animal facility at the Institute of Neurobiology, University of Puerto Rico Medical Sciences Campus. Snails were maintained in aquaria at room temperature under a 12:12 light-dark cycle and fed lettuce *ad libitum*.

*B*. *glabrata* were exposed to *S*. *mansoni* miracidia at the Biomedical Research Institute (BRI, Rockville MD). Schistosome eggs were obtained from mouse livers and hatched using BRI protocols [37]. Snails were incubated with miracidia (target: 5 per snail) for two hours. Tissues from infected specimens were dissected and collected at the BRI at 20 days (prepatent) and 35 days (shedding) post-infection (dpi). Shedding was verified by exposure of snails to direct light.

### Electrophysiology

The cDNA encoding the *Bgl-*FaNaC sequence was codon-optimized for *Xenopus laevis* and synthesized by Integrated DNA Technologies (IDT, Coralville IA). The Flag-tag protein affinity sequence (DYKDDDDK; Sigma-Aldrich) was added to the N-terminal to enable confirmation of expression. The cDNA was subcloned into the *Xenopus* expression vector pGEM HE as described previously [38]. The *Bgl-*FaNaC full-length RNA was transcribed *in vitro*, capped and polyadenylated using the T7 mScript Standard mRNA Production System (CellScript, Madison WI).

Oocytes were obtained from dissected ovaries of adult *Xenopus laevis* specimens (Xenopus Express, Brooksville FL). They were dispersed with type II collagenase and manually defolliculated. RNA injections were performed in pre-selected oocytes from stages V and VI. Oocytes were injected with 38.6 nL of *Bgl-*FaNaC encoding RNA at a concentration of 20 ng/uL using a Nanoliter 2000 microinjector (World Precision Instruments, Sarasota FL).

Protein expression was monitored daily for six consecutive days. Eight oocytes were homogenized (83 mM NaCl, 1 mM MgCl_2_, 10 mM HEPES, 5 mM EDTA, pH 7.9) using a glass micro-homogenizer. Homogenates were centrifuged at 14,000 G (4°C) and supernatants were stored at -80°C until needed. Protein lysate quantification was performed with the Precision Red Advance Protein Assay (Cytoskeleton Inc., Denver CO) following the manufacturer’s instructions. An equal amount (5 μL) of protein lysate was loaded on a 12% polyacrylamide gel. Following electrophoresis and transfer to a PVDF membrane, immunodetection was performed with an anti-Flag primary antibody diluted 1:200 in blocking solution (4°C, overnight rocking). Membranes were washed in TBS-T (3 x 10 min) and incubated in goat-anti rabbit peroxidase conjugated second anti body (1:4000; 1 h). Membranes were then washed with Tris-buffered saline, 0.01% Tween 20 (TBS-T; 3 x 10 min) and processed for chemiluminescent signal detection using the Super Signal West-Femto Maximum Sensitivity Substrate kit (Thermo Fisher Scientific, Waltham MA) following the manufacturer’s instructions. Maximum expression levels were observed 6 days following injection.

Injected oocytes were placed at the bottom of a plastic recording chamber (3 ml volume) lined with a nylon mesh and continuously perfused with ND96 (96 mM NaCl, 2 mM KCl, 1.8 mM CaCl_2_, 1 mM MgCl_2_, 5 mM HEPES, pH 7.6; see detailed methods in [38]). An OC725B oocyte clamp (Warner Instruments LLC., Hamden CT) was used to clamp the membrane potential at -60 mV with independent microelectrodes for recording (0.1 M potassium chloride) and passing current (3 M potassium chloride). Data were acquired on a Digidata 1200 interface with Clampex (V.6) and analyzed with AxoScope 10.7 (Axon Instruments, Union City CA). All experiments were carried out at room temperature (20–25°C).

Synthetic FMRF-NH_2_, FLRF-NH_2_, FIRF-NH_2_, pQFYRI-NH_2_, and GDPFLRF-NH_2_ peptides (>90% purity) were purchased from GL Biochem (Shanghai, China). All peptides were dissolved in ND96 in a stock of 1 mM. Peptide stocks were frozen at -20°C and serial dilutions were freshly prepared before each experiment. Peptides were applied manually using a pipettor (1 mL, upstream from the oocyte).

### Antibody validation

**Fig 1:**
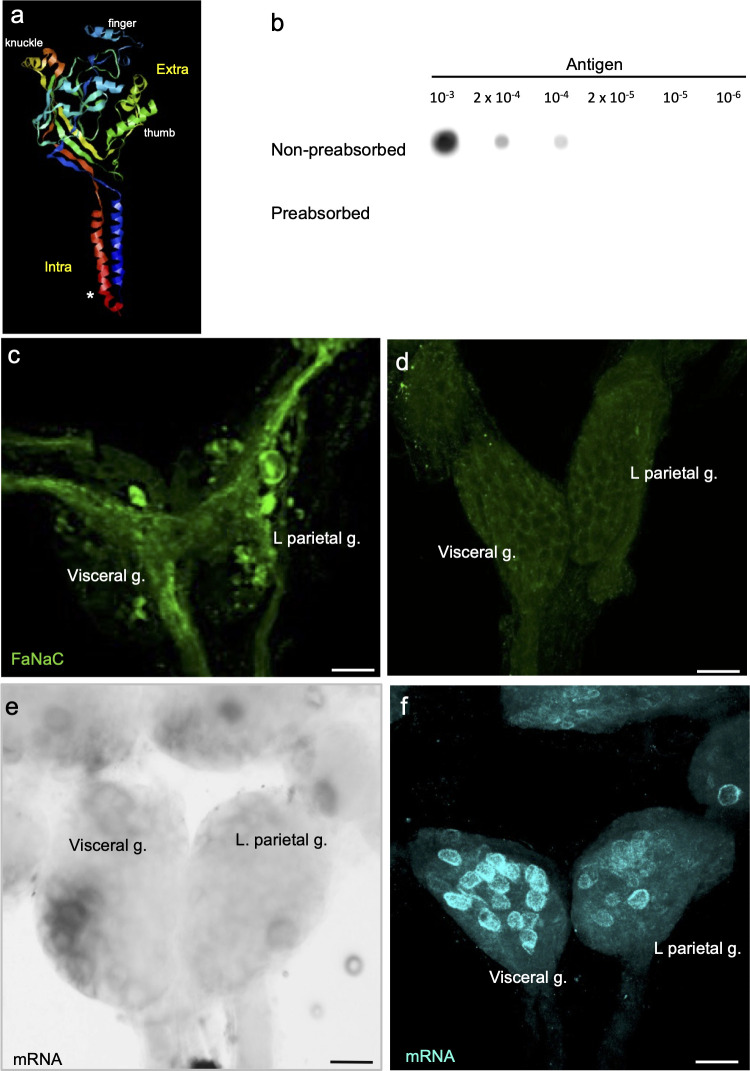
Localization of *Bgl-*FaNaC expression. **a:** Ribbon model of *Bgl-*FaNaC channel subunit produced with the Open RasMol Molecular Graphics Visualization Tool (v2.7; www.penrasmol.org). The channel comprises two intracellular (*Intra*) N- and C- termini and a large extracellular (*Extra*) domain. Features of the ‘clenched fist’ conformation are labeled (see [31]). The antibody used in this study was generated against a 14-residue domain near the amino terminus of the channel (*asterisk*). The selected sequence was based upon an analysis of antigenicity performed by GL Biochem (Shanghai, China). **b:** Dot blot controls demonstrate specificity of the *Bgl-*FaNaC antibody. Upper row: serial dilutions of a 2 μM antigen solution were blotted (2 μl) and probed with a 1:200 dilution of the antibody used in this study. Lower row: Preabsorption of the antibody with the antigen peptide (1 x 10^−4^ M, overnight) eliminated recognition of the blotted peptide. **c:** Wholemount immunolabeling of the ventral surface of the visceral and left parietal ganglia. Intense FaNaC labeling (*green*) was present in fiber systems coursing through the ganglia. **d:** FaNaC labeling was eliminated following antibody preabsorption with the antigen. **e:** The signal produced with the digoxygenin *in situ* hybridization protocol was confined to the cell bodies of neurons in the visceral and left parietal ganglia. **f:** Detection of FaNaC mRNA using the Hybridization Chain Reaction technique produced defined labeling (*cyan*) in the cell bodies of neurons in the visceral and left parietal ganglia. All calibration bars = 50 μm.

**Fig 2:**
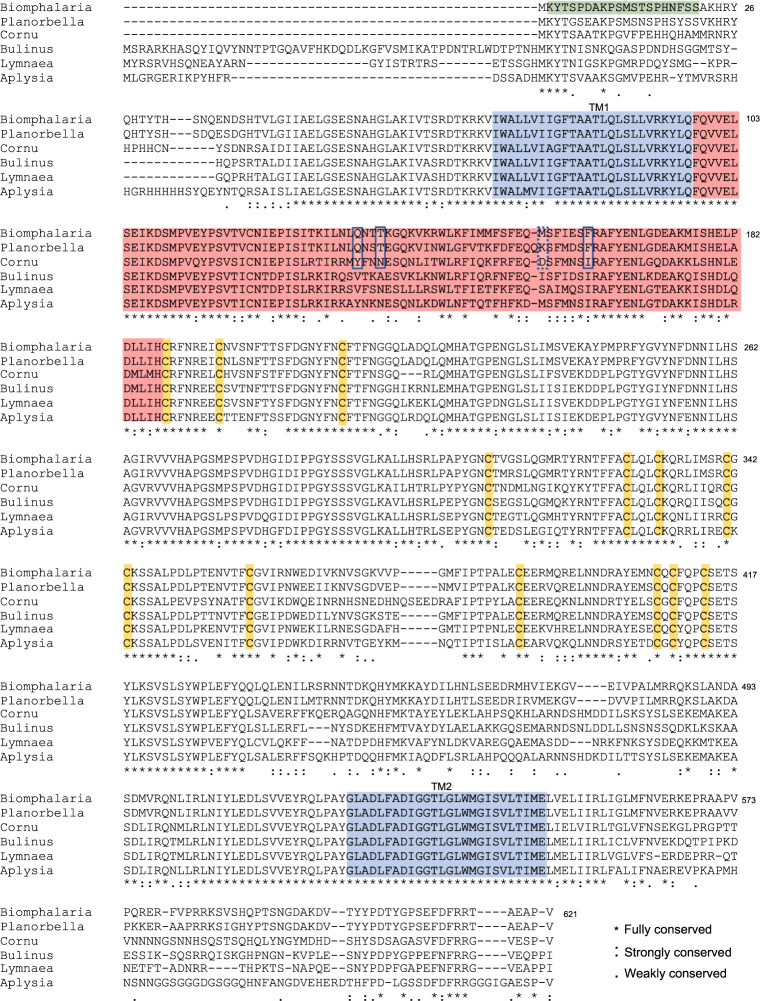
Sequence alignment of the *Bgl-*FaNaC with FaNaCs reported for other gastropods. Sequences include: *Planorbella trivolvis* (GenBank ID: AAF80601.1 [41]), C*ornu aspersum*, (GenBank ID: CAA63084.1; [25]), *Bulinus truncatus* (GenBank ID: KAH9494543.1), *Lymnaea stagnalis* (GenBank ID: AAK20896.1 [42]), and *Aplysia kurodai* (GenBank ID: AB206707.1 [43]). Amino acid numbering corresponds to *Bgl-*FaNaC. Sequence of the peptide used to generate the antibody used in this study is shaded light green. Orange shading highlights conserved cysteine residues that are thought to contribute to disulfide bridges in the finger region of the channel. Light blue shading denotes the two highly conserved transmembrane domains, TM1 and TM2. The less conserved region that is proposed to account for peptide recognition is shaded light red [44][45]. Within this domain, four specific residues that influence the concentration-response relationship are outlined (see [46]). The alignment was generated with the T-Coffee web server [47].

Affinity purified rabbit polyclonal antibodies were generated against the amino terminus intracellular domain of the *B*. *glabrata* FMRF-NH_2_-gated sodium channel (KYTSPDAKPSMSTS-C; residues 2–15, Figs 1A and 2) by GL Biochem Ltd., Shanghai, China (ELISA titer > 1:128,000). Solid phase specificity was confirmed with dot blots of serial antigen dilutions (2 μL) applied to nitrocellulose membrane (Bio-Rad 0.45 μm; Fig 1B). Membranes were allowed to air dry (1 h) and then incubated with blocking buffer (1 h, room temperature, shaking). Membranes were incubated overnight with the anti-FaNaC primary antibody diluted to 5 μg/ml in blocking solution (4°C, shaking). For preabsorption controls, the antibody was pre-incubated with the peptide antigen (10^−4^ M) overnight prior to application to the membrane. The membranes were then washed three times for 15 minutes and incubated with goat anti-rabbit IgG (H+L) second antibody conjugated to HRP (0.25 μg/ml in blocking solution, 1 h room temperature). They were then washed in TBS-T (4 x 15 min) and transferred to SuperSignal West Femto Maximum Sensitivity Substrate (Thermo Fisher Scientific, Waltham MA). The enzyme-substrate reaction was allowed to proceed for five minutes before visualization.

Preabsorption experiments on fixed *Biomphalaria* nervous tissue also verified the specificity of antigen detection. A 1:200 dilution of the *Bgl-*FaNaC antibody produced strong immunofluorescence (Fig 1C) that was eliminated when the antibody was preabsorbed with the antigen prior to tissue incubation (5 x 10^−4^ M, overnight; Fig 1D). Together, the preabsorption experiments supported the sensitivity and specificity of the antibody used in this study. They also provided guidance for primary and secondary antibody dilutions to use for antigen detection. Signals were eliminated when primary antibody incubation was omitted from the protocol (not shown).

### Wholemount immunohistochemistry

Standard wholemount immunohistochemical protocols were followed [39,40]. Tissues were dissected in normal saline (51.3 mM NaCl, 1.7 mM KCl, 1.5 mM MgCl_2_, 4.1 mM CaCl_2_, 5 mM HEPES, pH 7.8.), and pinned in a Petri dish lined with Sylgard (World Precision Instruments, Cat. No. SYLG184). Ganglia were incubated in protease (Type XIV, Sigma #P5147; 0.5% in normal saline; 7–10 min), washed thoroughly with normal saline, and fixed in 4% paraformaldehyde (1 h, room temperature).

Fixed tissues were washed 5 x 20 min in PTA (0.1 M phosphate buffer containing 2% Triton X-100 and 0.1% sodium azide) at room temperature. Samples were pre-incubated with normal goat serum (NGS; 0.8%, 3–12 h, room temperature) and then transferred to the primary antibody (5 μg/ml in PTA, 3–5 days). Samples were washed (5 x 20 min in PTA) and incubated in second antibodies conjugated to a fluorescent marker (Alexa 488 goat anti-rabbit IgG (H+L) conjugate; Molecular Probes, Eugene OR) at dilutions ranging from 1:500 to 1:1,000. No differences were evident across the ranges of NGS blocking times, antibody incubation durations, or second antibody concentrations. Results were assessed on a Nikon Eclipse epi-fluorescent microscope prior to confocal imaging on a Nikon A1R Confocal Laser Microscope using the NIS Elements AR software package. Image processing and analysis were performed with Fiji (NIH, GitHub open source) and figures were prepared with Microsoft PowerPoint (v.16.69.1).

### *In situ* hybridization

#### Wholemount *in situ* hybridization with chromogenic detection

Digoxygenin (DIG)-labeled probes were produced with the SuperScript III One-Step RT-PCR kit (Sigma 12574–026) using specific primers for the *Bgl-*FaNaC transcript (Forward: CCAGCATGTCTACCTCACCGCAC, Reverse: CTCCGTAGGCAAGTCCGGCAAGG). T7 and T3 promoter sequences were appended to the forward and reverse primers, respectively. The expected amplicon length was 1,034 bp. Ganglia were dehydrated and rehydrated in methanol/10x PBS graded solutions (25%, 50%, and 75% methanol) for 5 min and then rinsed in PBST (1% Tween 20) for 5 min. Tissues were digested with 2 mg/ml of Proteinase K (Thermo Fisher Scientific, Waltham MA) for 7 min, and fixed with 4% paraformaldehyde (room temperature, 45 min). After rinsing 5 × 5 min in blocking buffer (1 x PBS, 0.1% Tween 20, 0.1% BSA, 1% DMSO), ganglia were pre-hybridized in hybridization mix (50% formamide, 5 x SSC, 1 mg / ml yeast RNA, 50 μg/ml heparin, 0.1% Tween 20, 5 mM EDTA, 9 mM citric acid, in DEPC treated water) for 4–6 h. Hybridization was performed with the pre-heated antisense probe (final concentration = 1 ng/μl) overnight at 65°C. Tissues were washed with a graded series of hybridization mix solutions (75%, 50%, 25%) in 2x SSC for 10 min each, followed by two 30 min washes with 0.2x SSC, all at 65°C. Tissues were then rinsed in 0.2x SSC graded solutions (75%, 50%, and 25%) in PBST for 10 min each at RT. For detection, specimens were pre-incubated in blocking buffer for 4–6 h and then incubated in anti-DIG antibody conjugated to alkaline phosphatase (1:3000 blocking buffer, overnight). Finally, tissues were rinsed in PBST 6 × 15 min followed by two 5 min washes in alkaline phosphatase buffer (100 mM Tris pH 9.5, 50 mM MgCl_2_, 100 mM NaCl, 0.1% Tween 20, levamisole). Signal development was performed with 100% BM-Purple (Roche) in the dark (Fig 1E).

#### Hybridization Chain Reaction (HCR) fluorescence *in situ* hybridization (FISH)

HCR RNA-FISH methods were adapted from the Molecular Instruments, Inc. (Los Angeles CA) protocols website (https://www.molecularinstruments.com/). The *B*. *glabrata* CNS was dissected in normal saline and pinned on Sylgard-lined plates. Tissues were exposed to protease (0.5%, Type XIV, Sigma) diluted in normal saline (7–10 min) and fixed in 4% paraformaldehyde overnight. The CNS was washed 5 times for 15 minutes each with PTwA (0.1 M phosphate buffer containing 2% Tween 20 and 0.1% sodium azide). Samples were pre-hybridized in hybridization buffer (Molecular Instruments, Inc.) for 30 minutes, and then hybridized overnight at 37°C with a probe set generated for the *Bgl-*FaNaC transcript (FaNaC/ LOT PRI987). Multiplexed detection of transcripts was achieved with a cocktail of probe sets that also included the *Bgl-*FaRP1 precursor (LOT PRI087; 361 nucleotides, coding sequence, excluding the sequence shared with *Bgl-*FaRP2; see [35]) and the *Bgl-*FaRP2 precursor (LOT PRI298; 487 nucleotides, coding sequence, excluding the sequence shared with *Bgl-*FaRP1). Probes were diluted in hybridization buffer at a final concentration of 4 nmol/μL each. Samples were washed 4 x 15 minutes with wash buffer (Molecular Instruments, Inc.) and incubated at room temperature with amplification buffer (Molecular Instruments, Inc.) for 30 minutes. Aliquots of the hair pin amplifiers (h1 and h2; 5 μL each, from 100 μM stock) were heated (95°C for 90 s) and then placed in a dark box for 30 minutes at room temperature. Following cooling, 5 μL of each hair pin was added to 250 μL of amplification solution. Tissues were transferred to the amplification solution and incubated overnight at room temperature in a dark box. The following day, samples were washed 5 times (10 minutes each) with 5x SSCT (sodium chloride-sodium citrate buffer, 0.1% Tween) at room temperature. Image acquisition, image analysis, and figure preparation were performed as described above (Wholemount immunohistochemisty).

### Quantification of expression

As the fluorescence mRNA detection provided superior clarity and definition (Fig 1E and 1F), all expression measurements were obtained from samples using the HCR method. Images from control and infected samples were obtained using identical settings on the NIS Elements data acquisition program. Labeled neurons on the dorsal and ventral surfaces of each ganglion were counted using the Fiji image processing package (ImageJ.org) by an experimenter blinded to the treatment. The mean gray value cut-off for positive expression was set at 15. Neurons with a diameter less than 10 μm were excluded from the analysis. Overall mean gray values were obtained from a region of interest (ROI) demarcated with the Fiji “free hand selection” tool. For analysis of *Bgl-*FaRP1 and *Bgl-*FaRP2, specific clusters (B group, F group and E group; see [35]) were selected as the ROI for gray value measurements. As *Bgl-*FaNaC expression was more dispersed, the perimeter of each ganglion was traced to define the ROI.

### Statistical analysis

Data are presented as mean ± standard error of the mean (SEM). Individual measurements are plotted for each condition in the bar graphs. Statistical significance was determined with the Brown-Forsythe and Welch one-way analysis of variance (ANOVA) and Dunnett’s multiple comparison *post hoc* test. Tests were performed and graphs were generated with GraphPad Prism version 9.2.0.

## Results

### The *B*. *glabrata* FaNaC structure and function

A transcriptome generated from twelve pooled *B*. *glabrata* central nervous systems [35] yielded a transcript encoding the *B*. *glabrata* FMRFamide-activated amiloride-sensitive sodium channel-like protein previously derived from genomic sequence [28]. This 4444 nucleotide transcript (GenBank Accession number OP066530) included a 1395 nucleotide 5’ untranslated sequence, an open reading frame (ORF) encoding a 621 amino acid protein termed *Bgl-*FaNaC, and a 1186 nucleotide 3’ untranslated sequence. The *Bgl-*FaNaC amino acid sequence was identical to that deduced from the genome (GenBank Accession Number XP_013063507; [28]).

Alignment of the *Bgl-*FaNaC amino acid sequence with known heterobranch FaNaCs confirmed significant sequence identity (Fig 2; *Planorbella trivolvis*: 91% [41], *Bulinus truncatus*: 70%, *Lymnaea stagnalis*: 70% [42], *Aplysia californica*: 67% [43], *Cornu aspersum*: 66%). A ribbon model generated with the Open RasMol Molecular Graphics Visualization Tool (Fig 1A) retained several conserved characteristics of the DEG/ENaC ion channel superfamily, including two highly conserved transmembrane domains (Fig 2, shaded blue), intracellular N- and C- termini, and a large “clenched fist” ectodomain. Thirteen cysteine residues located in the finger region were fully conserved (Fig 2, orange shading). Lower conservation was observed in the thumb region, which is thought to confer agonist specificity and efficacy of FaNaC receptors [44,45,46].

**Fig 3:**
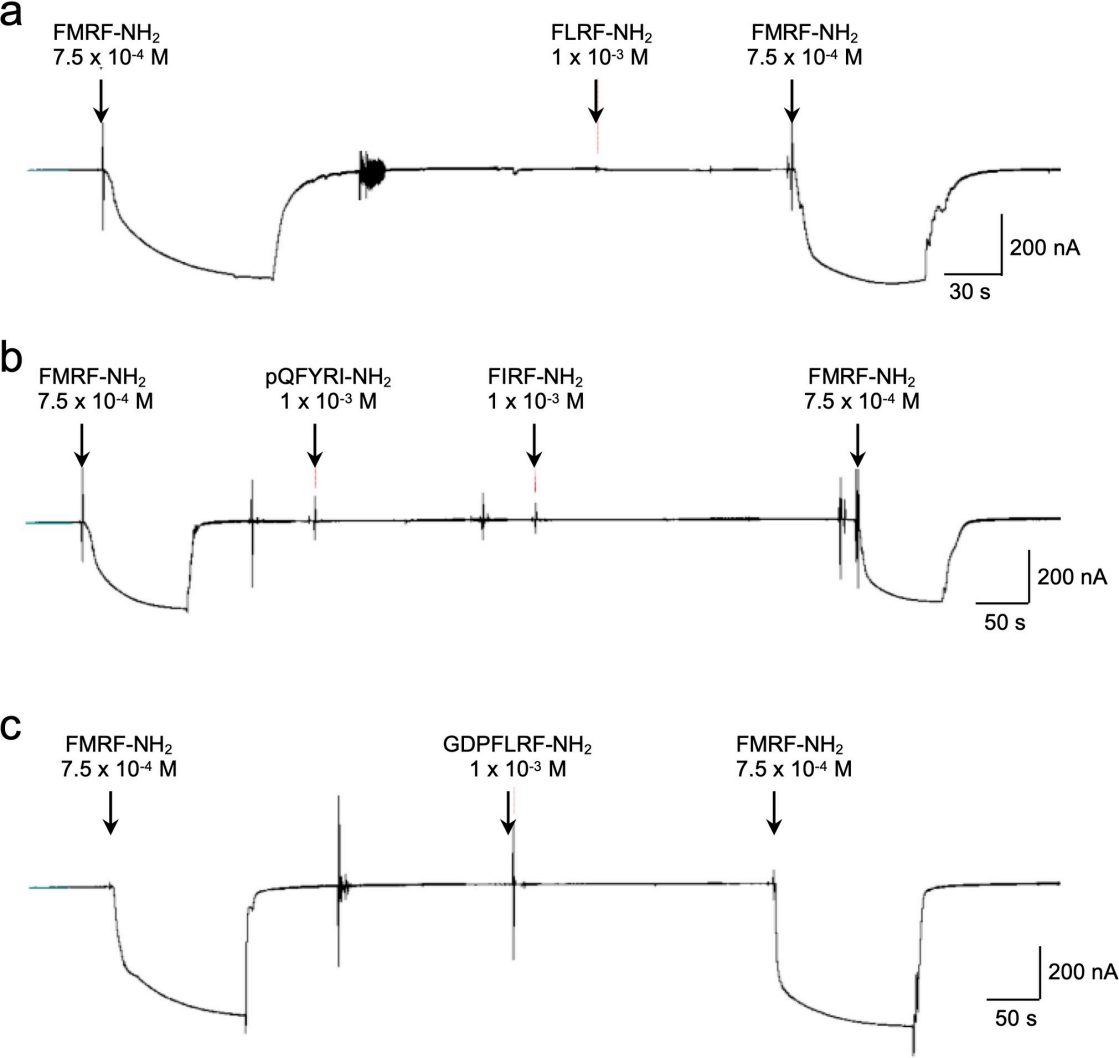
*Bgl-*FaNaC specificity demonstrated with heterologous expression in *Xenopus* oocytes. Currents were recorded with a two-electrode voltage clamp configuration. **a:** Application of FMRF-NH_2_ (7.5 x 10^−4^ M, 1 mL) produced large inward currents. No response was detected following application of FLRF-NH_2_ (1 mM). **b:** Tests with two peptides encoded on the FMRF-NH_2_ tetrapeptide precursor *Bgl-*FaRP1 [35], pQFYRI-NH_2_ (1 mM) and FIRF-NH_2_ (1 mM), substantiated the specificity of the *Bgl-*FaNaC. **c:** No response was elicited by GDPFLRF-NH_2_, a product of the heptapeptide precursor *Bgl-*FaRP2 produced by alternative splicing of the FMRF-NH_2_ message.

**Fig 4:**
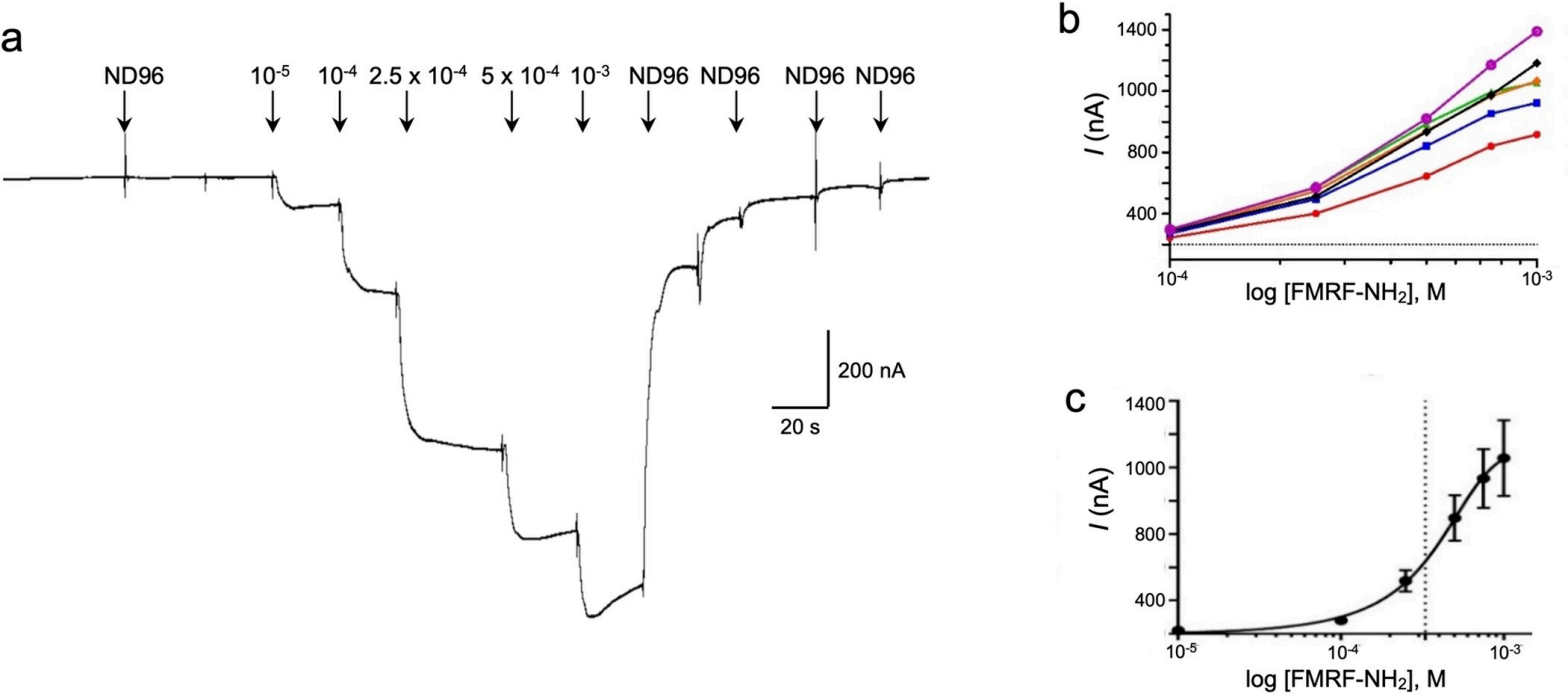
Concentration dependence of *Bgl-*FaNaC responses to FMRF-NH_2_. **a:** Application of graded concentrations of FMRF-NH_2_ (10^−5^ M– 10^−3^ M; 1 mL) produced increased inward currents in the *Xenopus* heterologous expression system. **b:** Uniform concentration-response profiles were obtained from five oocytes (color coded). **c:** Averaged data produced an EC_50_ of approximately 3 x 10^−4^ M (dashed line).

The *Bgl-*FaNaC coding sequence was optimized for expression in *Xenopus*, cloned into an expression vector, and injected into oocytes. Maximal expression was measured following six days (see Materials and Methods). Bath application of FMRF-NH_2_ (7.5 x 10^−4^ M; 1 mL) upstream from an oocyte produced an inward current (Fig 3A–3C). No currents followed application of related peptides that are encoded on the *Biomphalaria* FMRF-NH_2_ precursors [35], including FLRF-NH_2_ (Fig 3A), pQFYRI-NH_2_ (Fig 3B), FIRF-NH_2_ (Fig 3B), and the heptapeptide GDPFLRF-NH_2_ (Fig 3C). The FMRF-NH_2_ responses were concentration-dependent, with a mean EC_50_ of 3.3 x 10^−4^ M (Fig 4). Desensitization was not observed under the conditions of peptide delivery [24,26,48].

### *Bgl-*FaNaC localization

**Fig 5:**
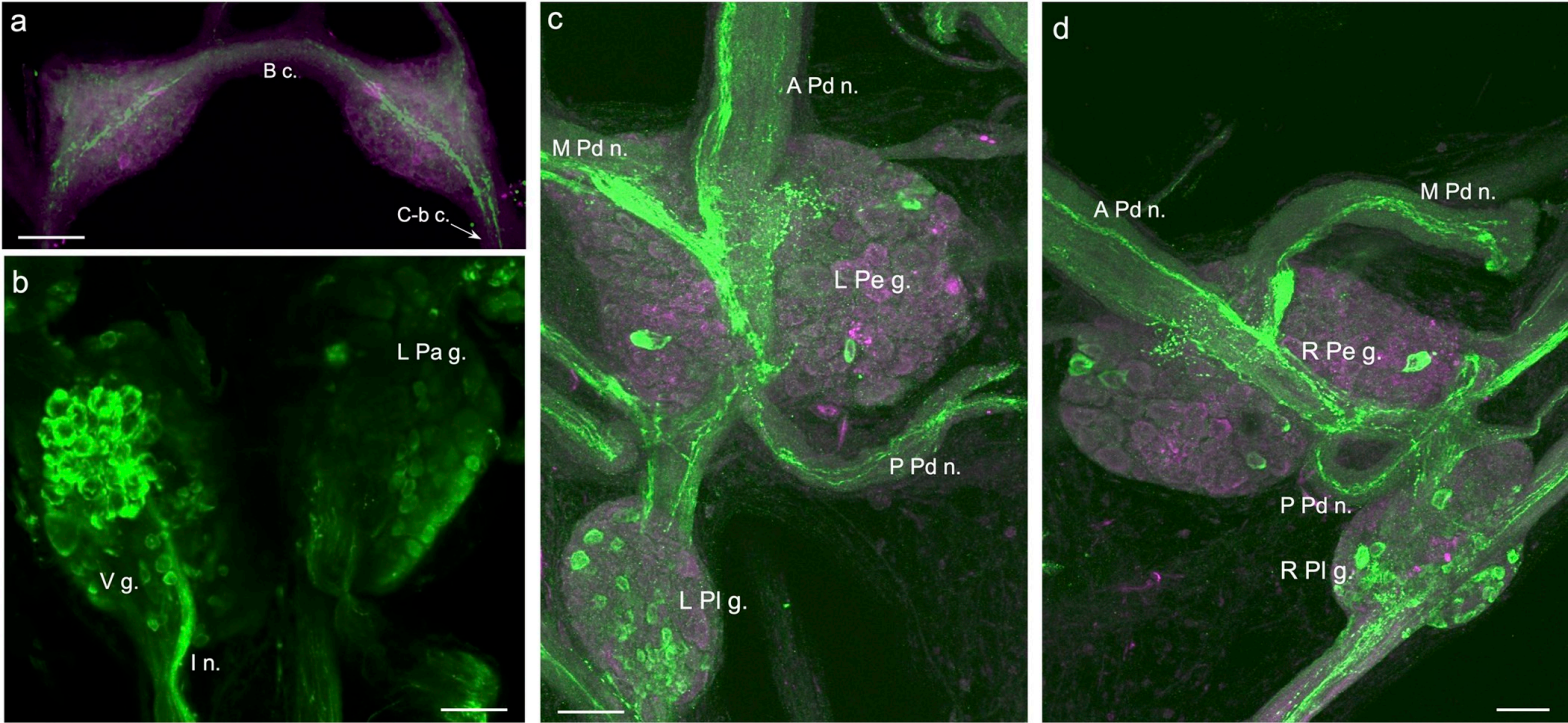
FaNaC-like immunoreactivity in the CNS of *Biomphalaria*. **a:** FaNaC-li fibers originating from the cerebral-buccal connective (*C-b c*.) crossed the buccal commissure (*B c*.). They coursed through each buccal hemiganglion giving rise to a diffuse network that permeated the central neuropil. *Calibration bar* = 50 μm. **b:** Intense labeling was observed in a cluster of cell bodies on the ventral surface of the visceral ganglion (*V g*.). These cells appeared to give rise to a compact bundle of fibers in the intestinal nerve (*I n*.). *Calibration bar* = 50 μm. **c:** FaNaC-li fibers coursed through the center of the left pedal ganglion (*L Pe g*.), projecting into the anterior, medial, and posterior pedal nerves (*A Pd n*., *M Pd n*., *P Pd n*.). Dispersed small neurons were located in the left pleural ganglion (*L Pl g*.). Dorsal surface shown. *Calibration bar* = 50 μm. **d:** FaNaC-li fibers coursed through the center of the right pedal ganglion (*R Pe g*.), projecting into the anterior, medial, and posterior pedal nerves (*A Pd n*., *M Pd n*., *P Pd n*.). Small neurons were located in the right pleural ganglion (*R Pl g*.). Dorsal surface shown. In panels **a**, **c**, and **d**, retrograde nerve tracing (magenta) was performed prior to the immunohistochemical protocol. *Calibration bar* = 50 μm.

**Fig 6:**
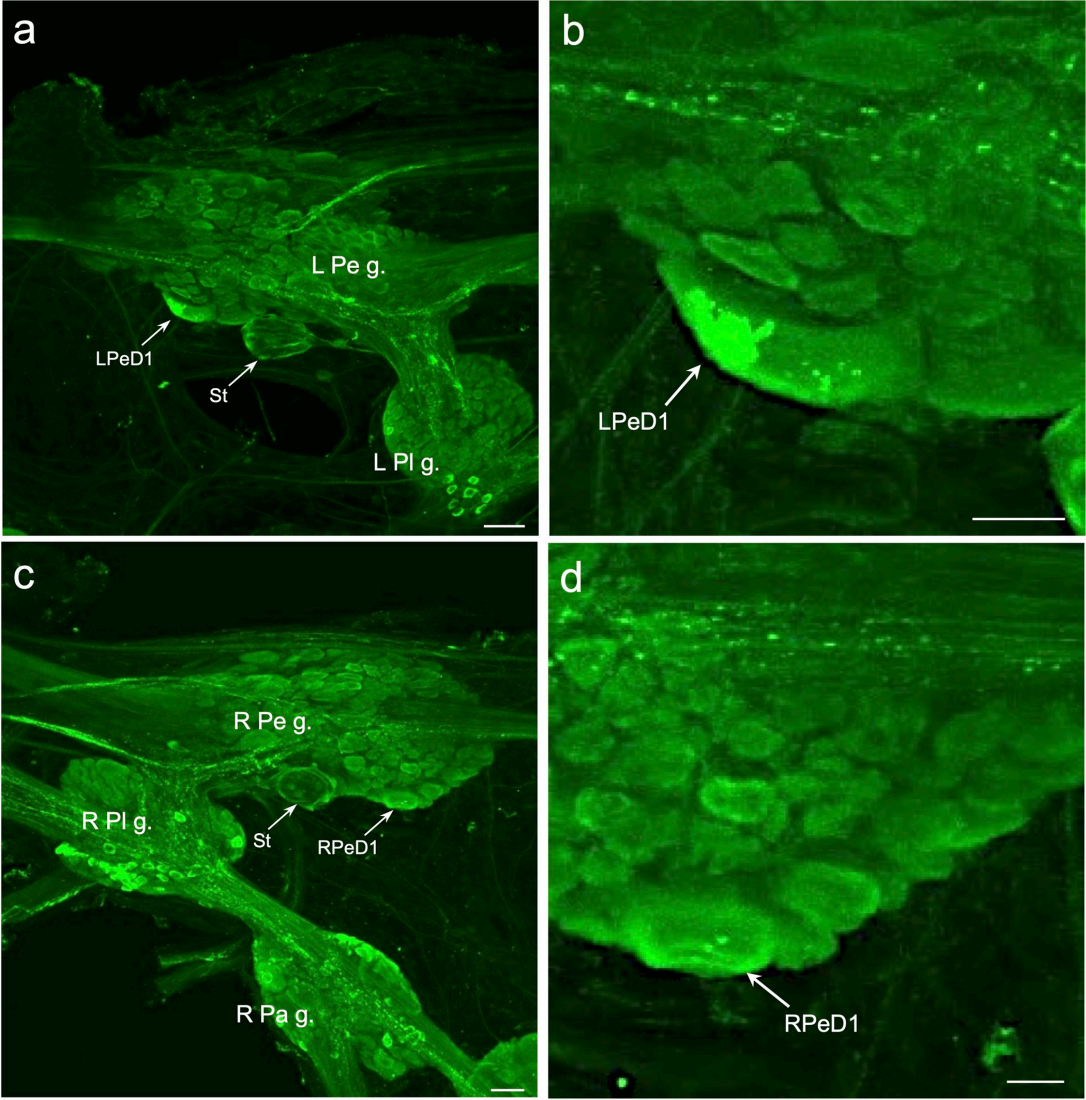
FaNaC-li in giant pedal ganglion neurons. **a:** The giant dopaminergic left pedal dorsal 1 (*LPeD1*) neuron was often optimally viewed from the ventral aspect of the ganglion (see Vallejo et al. 2014). FaNaC-li labeling was present in the LPeD1 and in the periphery of the statocyst (*St*). *Calibration bar* = 50 μm. **b:** Higher magnification revealed intense labeling of LPeD1 in a discrete region of the cell. *Calibration bar* = 20 μm. **c:** The giant right pedal dorsal 1 (*RPeD1*; [50]) cell was apparent on the ventral aspect of the ganglion. The periphery of the right statocyst (*St*) was also labeled. *Calibration bar* = 50 μm. **d:** With higher magnification, FaNaC-li labeling of the RPeD1 was more distributed than in LPeD1. *Calibration bar* = 20 μm.

A polyclonal rabbit antibody was generated against residues 2–15 of the *B*. *glabrata* FaNaC (Figs 1A and 2). Immunohistochemical processing of wholemount central nervous systems labeled a widespread network, with cell bodies located in all ganglia, and abundant fiber systems within the peripheral nerves (Figs 5 and 6). Dense fiber tracts also coursed through the central neuropil of the ganglia, often obscuring underlying cell bodies (Figs 1C and 5).

In the pedal ganglia, immunohistochemical FaNaC labeling was detected in two specific giant neurons, the dopaminergic left pedal dorsal 1 (LPeD1, Fig 6A and 6B; see [49]) and the serotonergic right pedal dorsal 1 (RPeD1, Fig 6C and 6D; see [50]). As reported previously in *Planorbella* [51], FaNaC labeling of the LPeD1 cell body appeared to be spatially aggregated (Fig 6B). FaNaC labeling of the RPeD1 cell body was more diffuse (Fig 6D).

**Fig 7:**
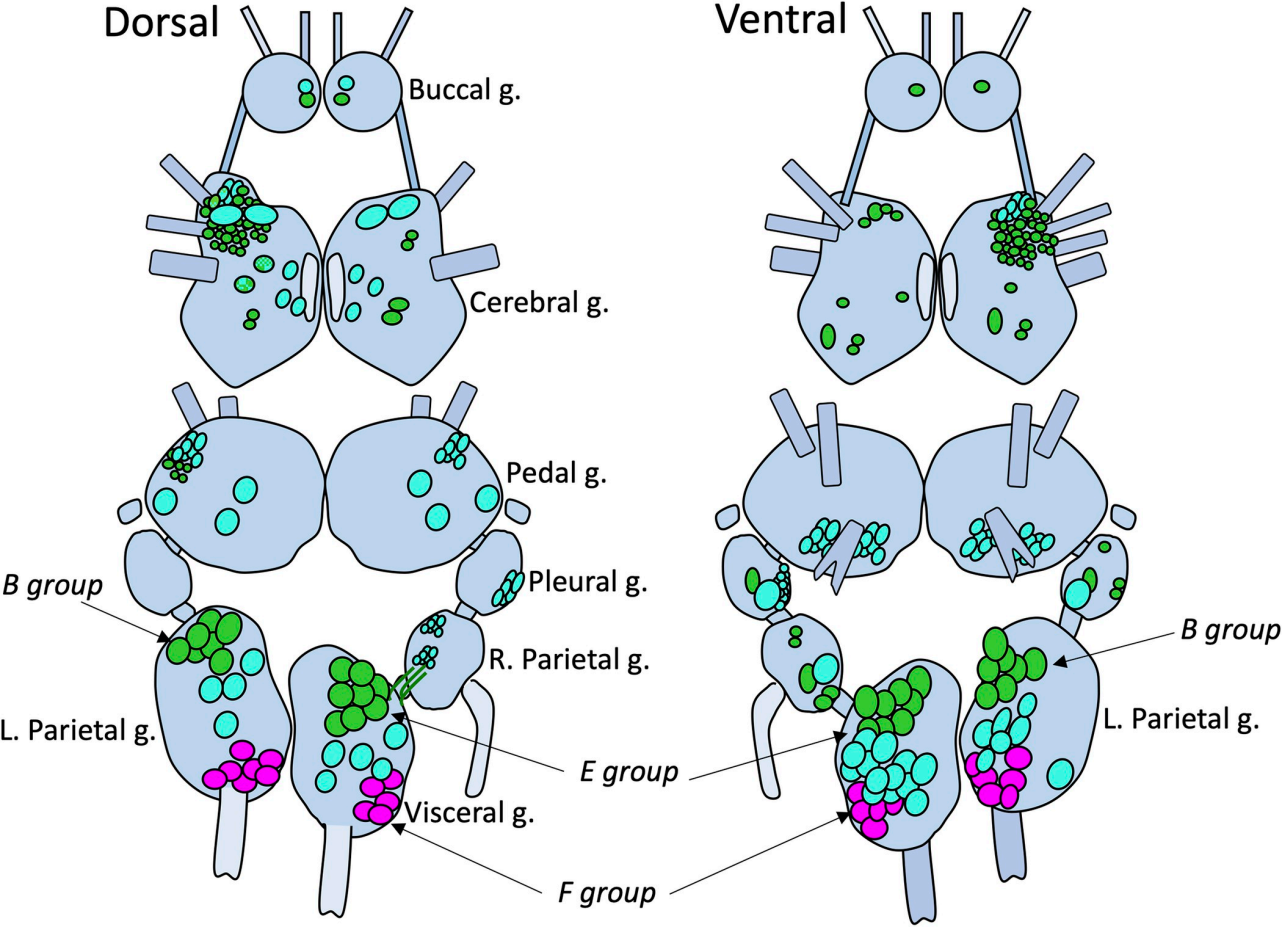
Schematic summary of expression patterns of the *B*. *glabrata* FaRP1 tetrapeptide precursor message (*green*), the FaRP2 heptapeptide precursor message (*magenta*), and the FaNaC receptor message (*cyan*) in the *B*. *glabrata* CNS. The paired buccal, cerebral, pedal, pleural, and parietal ganglia, and the unpaired visceral ganglion are labeled on the Dorsal schematic (left). Cell clusters relevant to this study include the B group in the left parietal ganglion and the E and F groups in the visceral ganglion.

**Fig 8:**
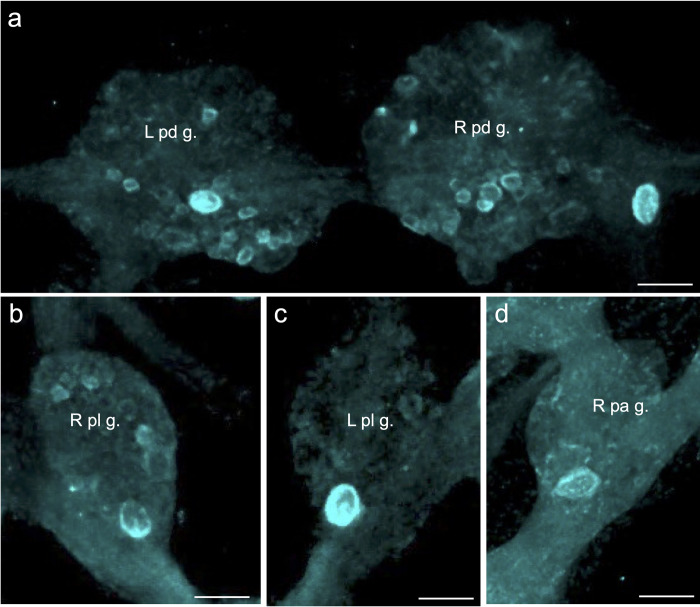
Large *Bgl-*FaNaC neurons labeled with HCR *in situ* hybridization. **a:** A single large neuron on the dorsal surface of each pedal ganglion was intensely labeled. Abbreviations: *L pd g*., left pedal ganglion; *R pd g*., right pedal ganglion. *Calibration bar* = 50 μm**. b:** Labeling was observed in one large neuron on the ventral surface of the right pleural ganglion (*R pl g*.). *Calibration bar* = 50 μm**. c:** One large neuron on the ventral surface of the left pleural ganglion (*L pl g*.) was intensely labeled. *Calibration bar* = 50 μm**. d:** *Bgl-*FaNaC mRNA was labeled in one large dorsal neuron in the right parietal ganglion (*R pa g*.). *Calibration bar* = 50 μm.

The two methods used to detect *Bgl-*FaNaC mRNA in wholemount nervous systems produced comparable results (see Materials and Methods). Chromogenic detection of cRNA probes (Fig 1C) and the fluorescent Hybridization Chain Reaction (HCR; Fig 1D) methods both produced labeling that was confined to cell somata, facilitating visualization of channel expressing cells (Fig 1E and 1F). *Bgl-*FaNaC expression assessed with the HCR method was widespread throughout the *B*. *glabrata* CNS (Fig 7, cyan). All eleven ganglia contained neurons expressing *Bgl-*FaNaC. Large intensely labeled cells were dispersed among the ganglia (Fig 8). A giant neuron was located in the inferior and lateral border of each pedal ganglion (Fig 8A). Each pleural ganglion contained one giant *Bgl-*FaNaC neuron (25–40 μm) near the ventromedial inferior border (Fig 8B and 8C). A single giant neuron in the ventromedial region of the right parietal ganglion was also labeled with HCR *in situ* hybridization for the *Bgl-*FaNaC transcript (Fig 8D).

**Fig 9:**
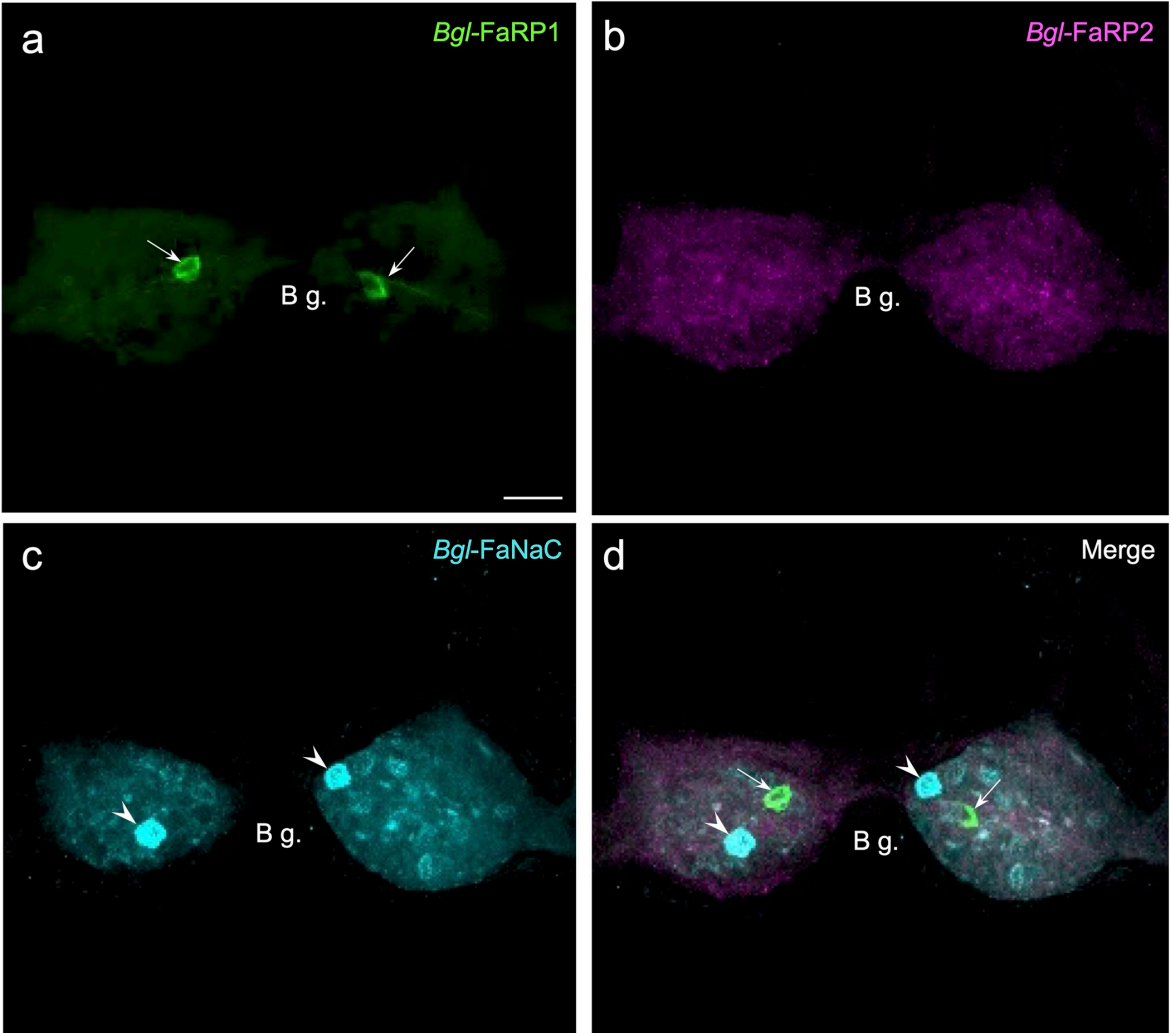
*Bgl-*FaNaC mRNA is not co-expressed with the FMRF-NH_2_ peptide precursors in the buccal ganglion. **a:** Tetrapeptide precursor *Bgl-*FaRP1 mRNA labeling was detected in two neurons in the buccal ganglia *(arrows*, see [35]). *Calibration bar* = 30 μm, applies to all panels. **b:** Expression of the heptapeptide *Bgl-*FaRP2 precursor was not observed in buccal neurons. **c:** Two buccal cells exhibited strong FaNaC mRNA labeling (*arrowheads*). **d:** An overlay of panels a-c showed that the cells expressing FaNaC (*arrowheads*) did not correspond to the neurons expressing FaRP1 (*arrows*).

The multiplexing capacity of the HCR system enabled comparison between *Bgl-*FaNaC localization and expression of the FaRP precursors. In agreement with previous immunohistochemical observations [35], strong expression of the tetrapeptide *Bgl-*FaRP1 precursor occurred in a single pair of neurons in the buccal ganglia (Figs 7 and 9A). The buccal ganglia were devoid of neurons expressing *Bgl-*FaRP2 (Figs 7 and 9B). While strong labeling of the *Bgl-*FaNaC receptor was also observed in a single pair of buccal neurons (Fig 9C), multiplexed hybridization showed that the receptor expression did not colocalize with its peptide agonist (Fig 9D).

**Fig 10:**
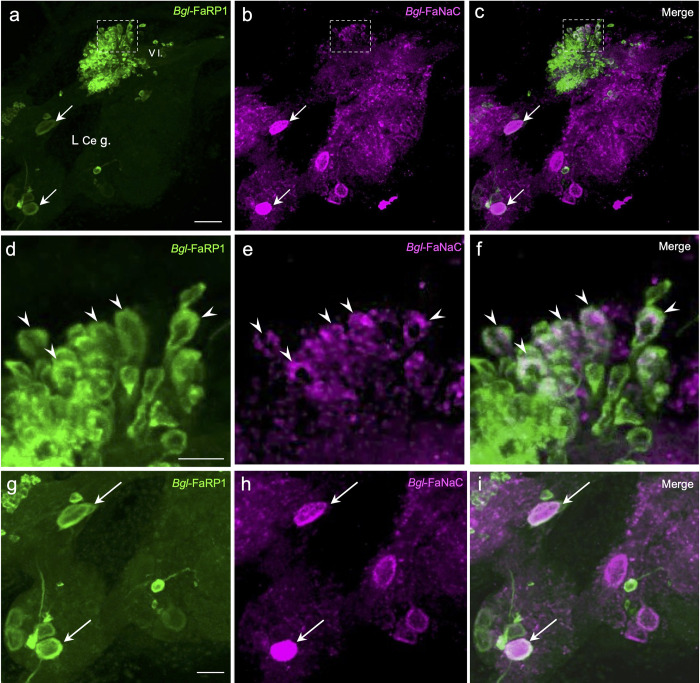
Colocalization of *Bgl-*FaNaC mRNA and *Bg*FaRP1 mRNA in neurons of the cerebral ganglion. Anterolateral quadrant of the left cerebral hemiganglion ventral surface shown. **a:** *Bgl-*FaRP1 mRNA was present in numerous small cells in the ventral lobe (*V l*.) of the left cerebral ganglion (*L Ce g*.; see [35]). *Calibration bar* = 30 μm, applies to **a-c**. **b:** A cluster of neurons in the lateral V l. express *Bgl-*FaNaC mRNA (*dashed rectangle*). Two larger neurons in the ventrolateral cerebral ganglion that express *Bg*FaRP1 also express *Bgl-*FaNaC (*arrows* in panels **a** and **b**). **c:** Overlay of panels **a** and **b** shows colocalization of *Bgl-*FaNaC mRNA and *Bg*FaRP1 mRNA in V l. cells. Neurons expressing both transcripts appear white. **d:** Region enclosed by *dashed rectangle* in **a** shown at higher magnification. Five neurons expressing *Bgl-*FaRP1 mRNA indicated by *arrowheads*. *Calibration bar* = 10 μm, applies to **d-f**. **e:** *Bgl-*FaNaC mRNA in the same field as **d**. Five labeled cells indicated by *arrowheads*. **f:** Overlay of panels **d** and **e** confirms co-expression of *Bgl-*FaRP1 and *Bgl-*FaNaC transcripts in a subset of neurons in the ventral lobe. **g:** Lower left quadrant of panel **a** shown at higher magnification. Two neurons expressing *Bgl-*FaRP1 mRNA indicated by *arrows*. *Calibration bar* = 20 μm, applies to **g-i**. **h:** *Bgl-*FaNaC mRNA in the same field as **g**. Two labeled cells indicated by *arrows*. **i:** Overlay of panels **g** and **h** confirms co-expression of *Bg*FaRP1 and *Bgl-*FaNaC transcripts in two anterolateral neurons extrinsic to the ventral lobe.

Colocalization of the *Bgl-*FaRP1 and *Bgl-*FaNaC messages was observed in the left ventral lobe (VL) of the cerebral ganglion, a lateralized CNS region involved in penile control (Fig 10; [35,52,53]. While the majority of VL neurons that expressed *Bgl-*FaRP1 did not label for *Bgl-*FaNaC, colocalization did occur in a cell cluster in the anterolateral region of the lobe (Fig 10D–10F). Colocalization of receptor and agonist expression was also observed in two larger left cerebral ganglion cells that were not within the VL (Fig 10A–10C and 10G–10I). These observations suggest that the *Bgl-*FaNaC could play a presynaptic or autoreceptor role in the circuits that control male reproductive behavior ([53], see Discussion).

**Fig 11:**
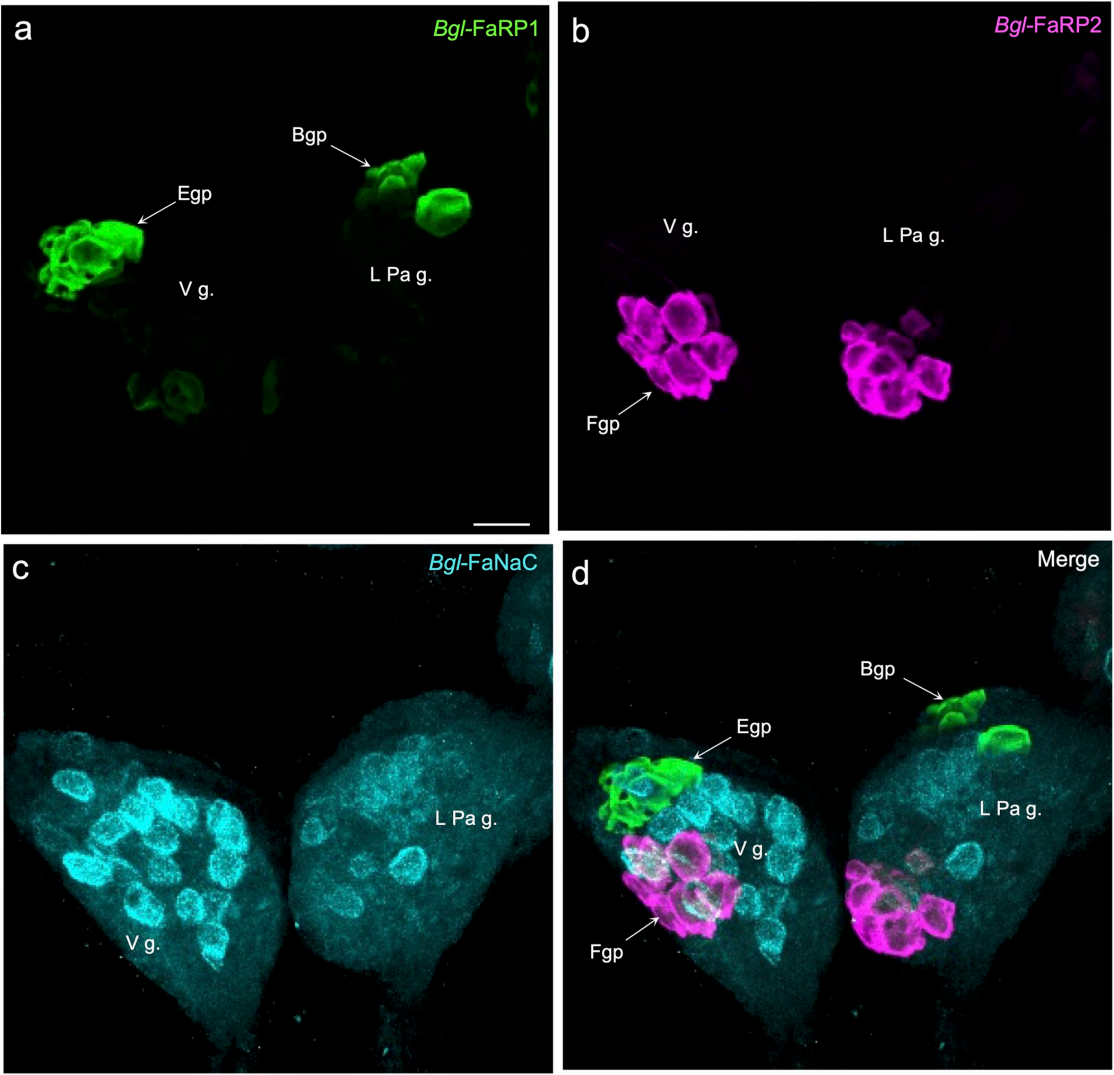
Distinct expression patterns of *Bgl-*FaNaC and the FMRF-NH_2_ precursors in the visceral and left parietal ganglia. **a:** Tetrapeptide precursor *Bg*FaRP1 mRNA labeling was detected in the anterolateral E group (*Egp*) of neurons in the visceral ganglion (*V g*.) and in the B group (*Bgp*) of anterolateral cells in the left parietal ganglion (*L Pa g*.). Ventral surface shown. *Calibration bar* = 50 μm applies to all panels. **b:** Expression of the heptapeptide *Bgl-*FaRP2 precursor was observed in the posterolateral F group (*Fgp*) in the visceral ganglion and in a posteromedial cluster of neurons in the left parietal ganglion. **c:** Cells labeled for FaNaC mRNA spanned the central region of the visceral ganglion. Labeling was less intense on the ventral surface of the left parietal ganglion where it also occupied the region between the peptide expressing clusters. **d:** Overlay of panels a-c showed that the cells expressing FaNaC intersected with the tetrapeptide and heptapeptide clusters, but co-expression of the receptor with the peptides was not detected in individual cells.

Localization of *Bgl-*FaRP1 and *Bgl-*FaRP2 expression in the visceral and left parietal ganglia agreed with immunohistochemical findings obtained with precursor specific antibodies [35]. Abundant *Bgl-*FaRP1 expression was observed in the anterolateral E group (Egp) of cells in the visceral ganglion and the B group (Bgp) of the left parietal ganglion (Figs 7 and 11A). The heptapeptide *Bgl-*FaRP2 precursor mRNA was expressed in the posterolateral F group (Fgp) of the visceral ganglion and in a posteromedial cluster in the left parietal ganglion (Figs 7 and 11B). *Bgl-*FaNaC was expressed in neurons spanning the region between the Egp and the Fgp on the ventral surface of the visceral ganglion (Fig 11C). Although a few of the *Bgl-*FaNaC cells overlapped with the E and F groups of the visceral ganglion, no co-expression of the peptide precursors and the receptor was detected (Fig 11D).

### FaRP precursor and *Bgl-*FaNaC expression following infection

**Fig 12:**
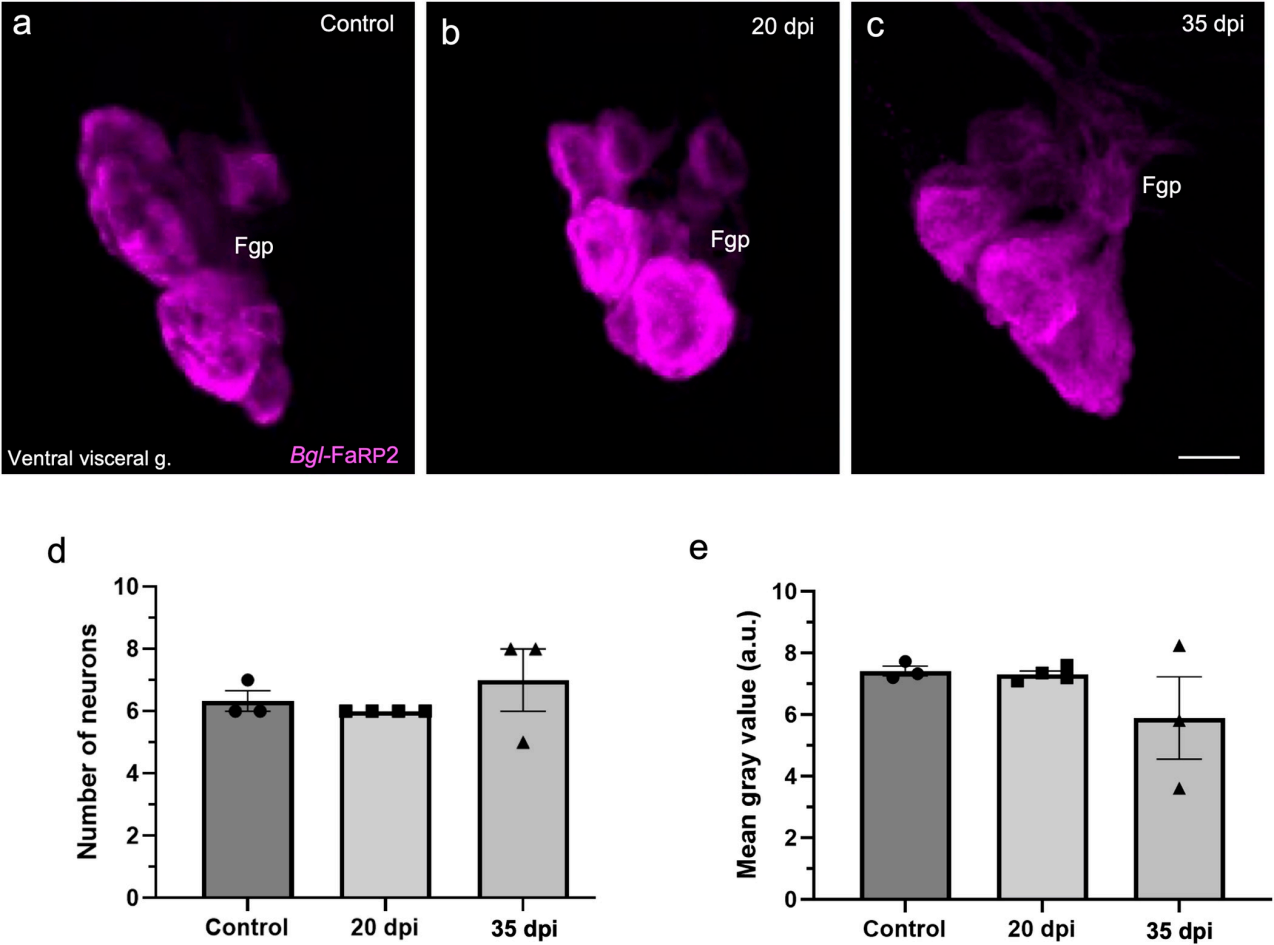
Expression of the heptapeptide *Bgl-*FaRP2 precursor in the visceral ganglion was not altered following *S*. *mansoni* infection. **a:** In uninfected specimens, HCR *in situ* hybridization for *Bgl*-FaRP2 labeled 6.3 ± 0.6 Fgp neurons cells with a mean grey value of 7.42 ± 0.28. **b, c:** Differences in expression were not observed at 20 or 35 dpi. *Calibration bar* = 30 μm. **d:** Summary data showed that the number of visceral Fgp neurons expressing *Bgl*-FaRP2 did not differ from control levels at the 20 or 35 dpi time points. **e:** Summary data confirmed that the mean gray values for *Bgl*-FaRP2 in the Fgp neurons did not differ from control levels at 20 or 35 dpi. (a.u.: arbitrary units). See Table 1.

**Fig 13:**
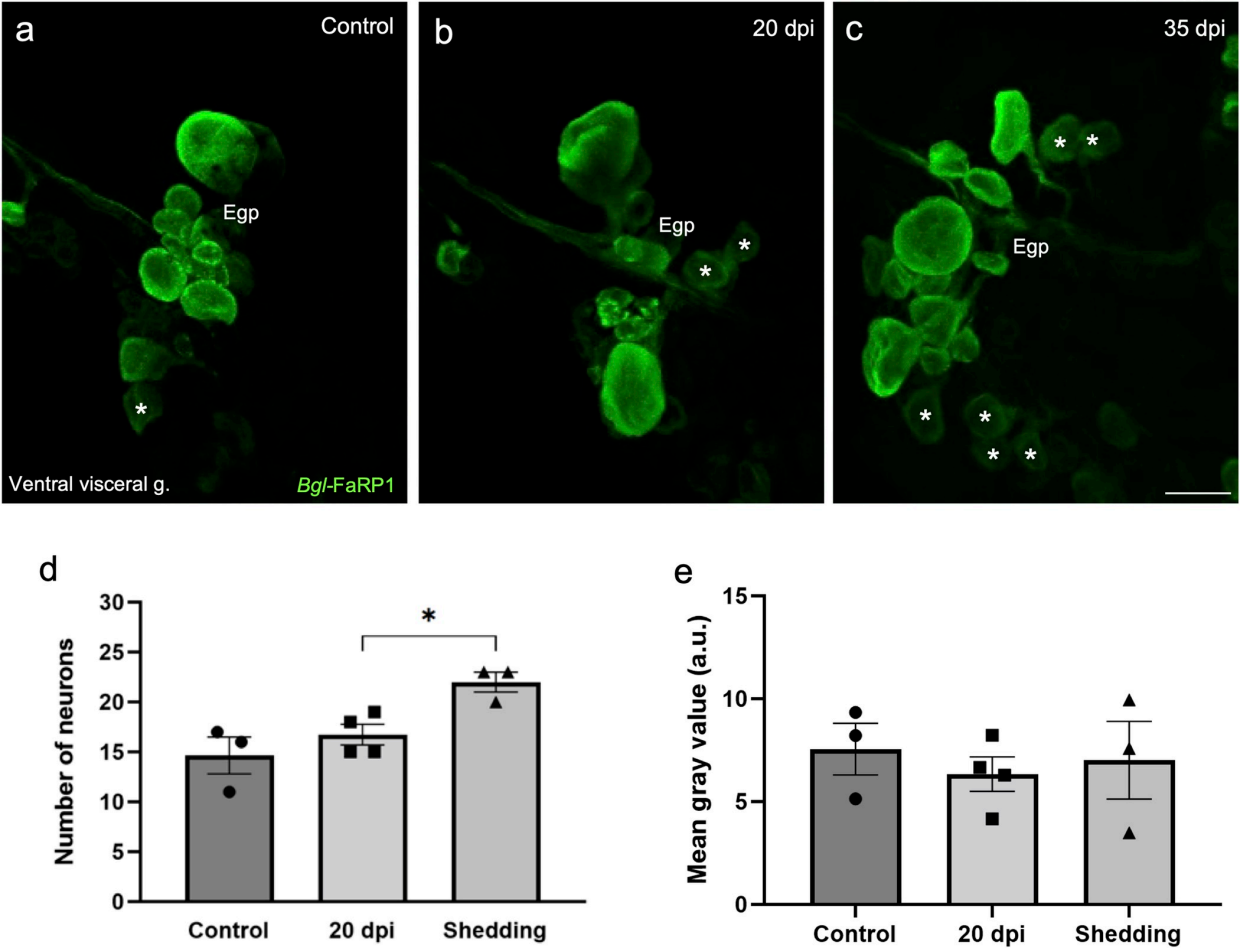
Expression of the tetrapeptide *Bgl-*FaRP1 precursor in the visceral ganglion was increased following *S*. *mansoni* infection. **a-c:** *Bgl-*FaRP1 mRNA detected with *in situ* hybridization in the Egp on the ventral surface of the visceral ganglion. *Calibration bar* = 30 μm, applies to a-c. **a:** In uninfected specimens, *Bgl*-FaRP1 expression in the visceral E group was detected in 14.7 ± 3.2 cells with a mean grey value 7.57 ± 2.17 (Table 1). **b:** No significant differences were observed in the number or intensity of labeled Egp cells at 20 dpi. **c:** At 35 dpi, the number of Egp neurons expressing *Bgl-*FaRP1 was increased. Asterisks indicate cells in which low levels of FaRP1 were detected. *Calibration bar* = 30 μm applies to all panels. **d:** Summary data confirmed that the number of Egp neurons expressing *Bgl*-FaRP1 was increased at 35 dpi. **e:** The mean gray value of visceral Egp neurons expressing *Bgl-*FaRP1 did not differ from control levels at 20 or 35 dpi. (a.u.: arbitrary units).

**Fig 14:**
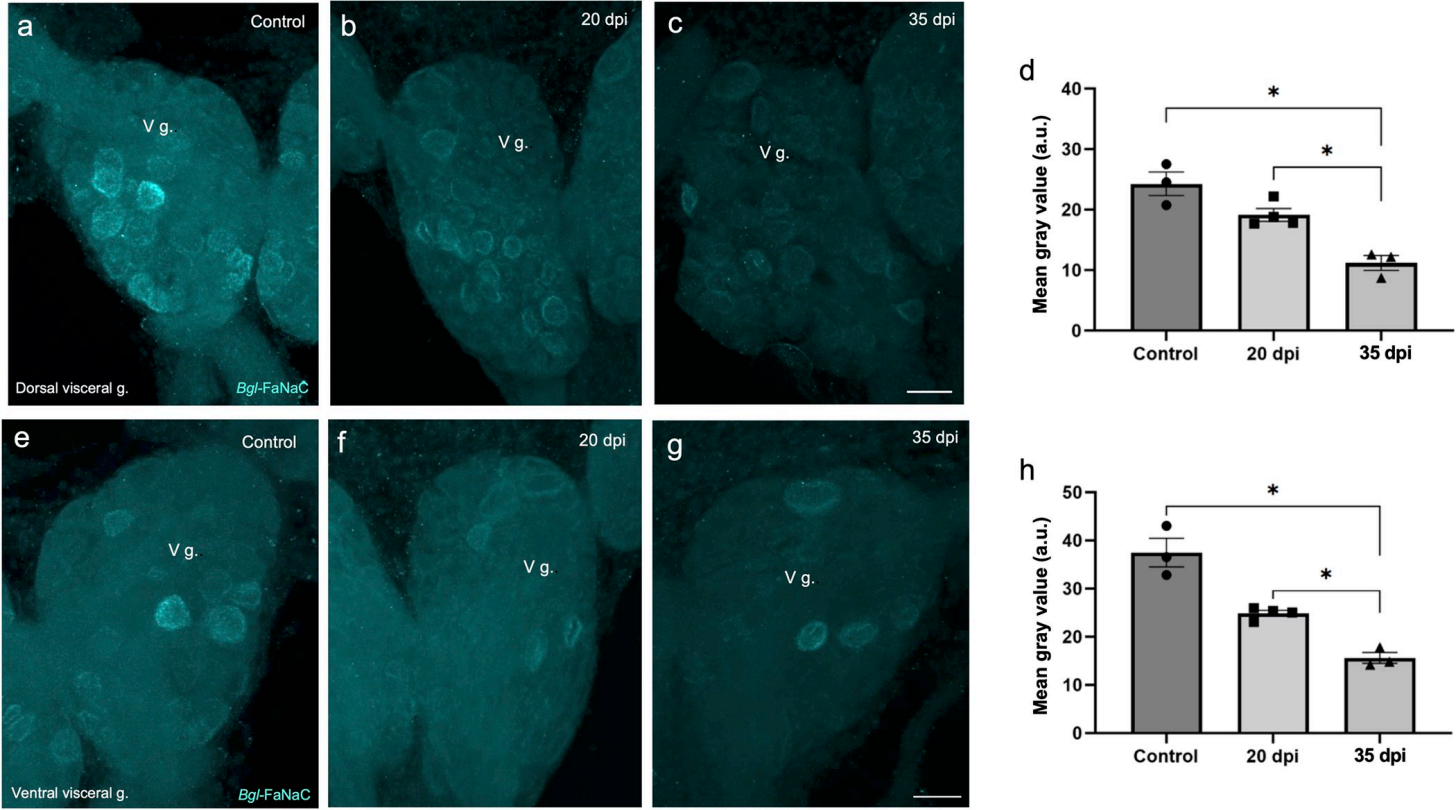
Decreased *Bgl-*FaNaC expression in the visceral ganglion following *S*. *mansoni* infection. **a-c:**Decreased *Bgl-*FaNaC hybridization on the dorsal surface of the visceral ganglion in infected specimens. *Calibration bar* = 30 μm, applies to a-c. **d:** Group data show a significant reduction in the mean grey value on the dorsal surface of the visceral ganglion at 35 dpi. Dunnett’s *post hoc* test: *, *p* < 0.05. See values in Table 1. **e-g:** Decreased *Bgl-*FaNaC hybridization on the ventral surface of the visceral ganglion in infected specimens. *Calibration bar* = 30 μm, applies to e-g. **h:** Group data show a significant reduction in the mean grey value on the ventral surface of the visceral ganglion at 35 dpi. Dunnett’s *post hoc* test: *, *p* < 0.05. See values in Table 1. (a.u.: arbitrary units).

**Fig 15:**
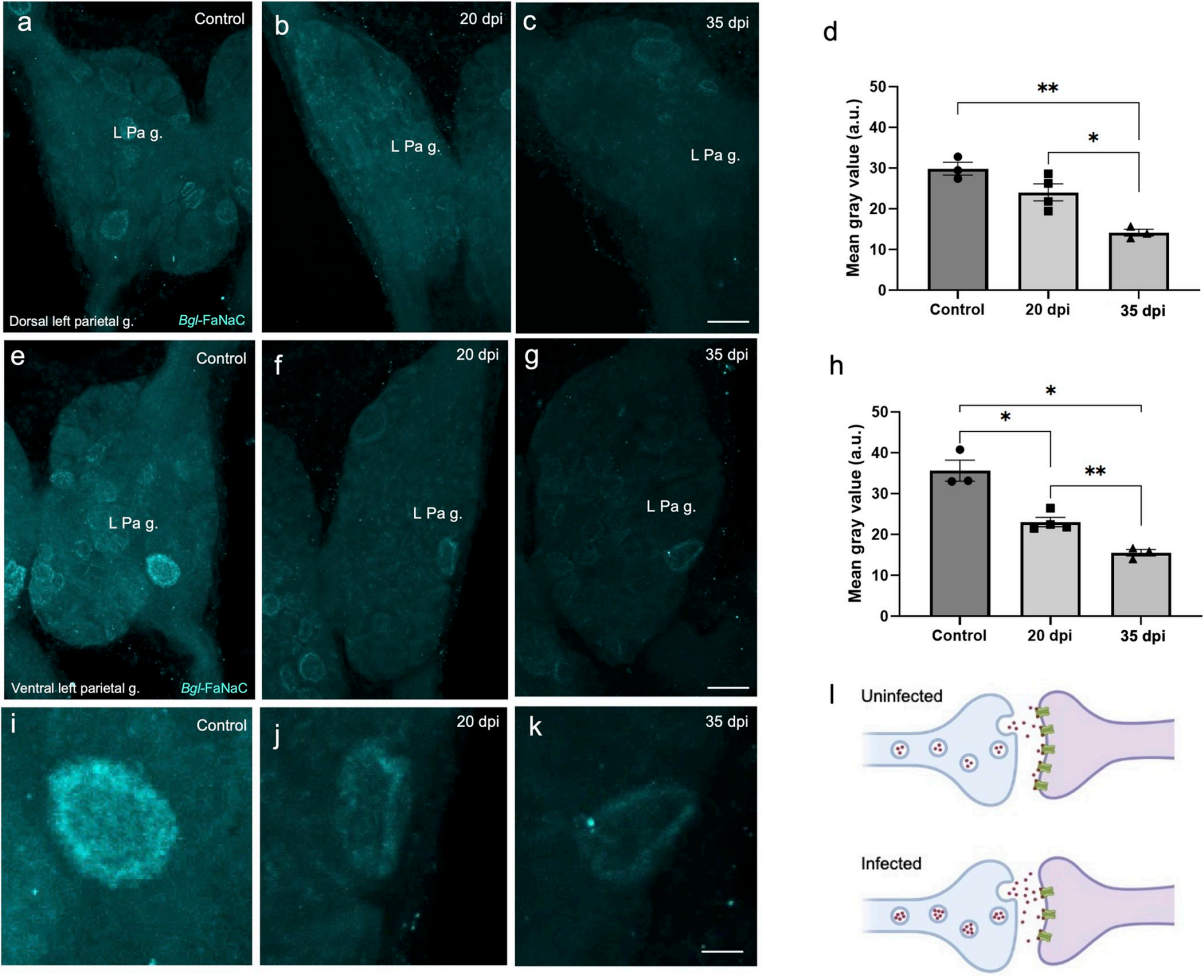
Decreased *Bgl-*FaNaC expression in the left parietal ganglion following *S*. *mansoni* infection. **a-c:** Decreased *Bgl*-FaNaC hybridization on the dorsal surface of the left parietal ganglion in infected specimens. *Calibration bar* = 30 μm, applies to a-c. **d:** Group data show a significant reduction in the mean grey value on the dorsal surface of the left parietal ganglion at 35 dpi. Dunnett’s *post hoc* test: *, *p* < 0.05. **, *p* < 0.01. See values in Table 1. **e-g:** Decreased *Bgl*-FaNaC hybridization on the ventral surface of the left parietal ganglion in infected specimens. *Calibration bar* = 30 μm, applies to e-g. **h:** Group data show a significant reduction in the mean grey value on the ventral surface of the left parietal ganglion at 20 dpi and at 35 dpi. Dunnett’s *post hoc* test: *, *p* < 0.05. **, *p* < 0.01. See values in Table 1. **i-k:** Large ventral left parietal ganglion neuron (panels e-g) shown at higher magnification. *Calibration bar* = 10 μm, applies to i-k. **l:** Proposed responses of *Bgl*-FaNaC synapses to *S*. *mansoni* infection. Increased expression of the FMRF-NH_2_ peptide agonist (red circles) is hypothesized to reflect a compensatory response to decreased *Bgl*-FaNaC expression (green receptors). Such opposing actions could exert a stabilizing influence on FMRF-NH_2_ ligand-gated signaling.

Due to the multiplexing capability and high resolution attained with the HCR protocol, this method was utilized for experiments testing potential effects of *S*. *mansoni* infection on peptide and receptor expression in the left parietal and visceral ganglia (Table 1 and Figs 12–15). Nervous systems were dissected from snails that were not exposed to miracidia, from size-matched specimens at 20 days post infection (dpi), and from ‘shedding’ snails at 35 dpi. Shedding was verified by stimulating release of cercariae upon exposure to light. Expression was quantified by counting the number of cells with HCR signals above background intensity levels and by measuring the average intensity of labeled cells (Table 1). Summary data showed that the number of cells expressing the *Bgl-*FaRP2 heptapeptide precursor was unchanged at the time points examined (Table 1 and Fig 12A–12D, visceral ganglion Fgp shown; control: 6.3 ± 0.6 cells; 20 dpi: 6.0 ± 0.0 cells; 35 dpi: 7.0 ± 1.7 cells; ANOVA: *F*_(2,7)_ = 0.742; *p* = 0.56). Mean gray values of the *Bgl-*FaRP2 HCR signals were also unchanged (Table 1 and Fig 12A–12C and 12E, visceral ganglion Fgp shown; control: 7.42 ± 0.28; 20 dpi: 7.31 ± 0.22;35 dpi: 5.89 ± 2.32; ANOVA: *F*_(2,7)_ = 1.182; *p* = 0.45 These findings indicate that expression of the FaRP heptapeptide (GDPFLRF-NH_2_) precursor is not affected by *S*. *mansoni* infection at the time points tested.

**Table 1:** Expression of FaRP precursors and *Bgl-*FaNaC in the left parietal and visceral ganglia of *B*. *glabrata*. Data were obtained from ganglia dissected 20 and 35 days post infection (dpi) with *Schistosoma mansoni*. Ganglia were processed with multiplexed HCR *in situ* hybridization (Fig 11D).

		Number of Neurons				Gray Value Intensity			
		Control	20 dpi	35 dpi	P value	Control	20 dpi	35 dpi	P value
*Bgl*-FaRP1									
Left Parietal									
	Dorsal	9.7 ± 2.3	5.5 ± 1.2	8.0 ± 3.6	*p* = 0.22	6.01 ± 0.51	7.96 ± 3.46	5.43 ± 0.73	*p* = 0.31
	Ventral	11.3 ± 2.3	16.0 ± 4.5	13.3 ± 0.6	*p* = 0.19	5.78 ± 2.50	6.72 ± 1.08	6.56 ± 1.31	*p* = 0.78
Visceral									
	Dorsal	10.7 ± 2.5	10.8 ± 4.6	14.7 ± 4.0	*p* = 0.37	4.29 ± 0.29	7.15 ± 3.63	5.84 ± 1.49	*p* = 0.33
	Ventral	14.7 ± 3.2	16.8 ± 2.1	22.0 ± 1.7^†^	*p* = 0.03	7.57 ± 2.17	6.34 ± 1.68	7.01 ± 3.27	*p* = 0.81
*Bgl*-FaRP2									
Left Parietal									
	Dorsal	6.7 ± 0.6	5.0 ± 1.4	3.7 ± 1.2	*p* = 0.04	7.58 ± 0.62	8.54 ± 1.54	7.41 ± 2.19	*p* = 0.53
	Ventral	6.7 ± 0.6	7.3 ± 0.5	8.7 ± 1.2	*p* = 0.09	6.60 ± 1.39	10.03 ± 2.41	8.82 ± 1.89	*p* = 0.09
Visceral									
	Dorsal	5.3 ± 0.6	5.3 ± 0.1	7.7 ± 1.5	*p* = 0.05	6.24 ± 2.45	7.70 ± 2.41	6.23 ± 1.26	*p* = 0.58
	Ventral	6.3 ± 0.6	6.0 ± 0.0	7.0 ± 1.7	*p* = 0.56	7.42 ± 0.28	7.31 ± 0.22	5.89 ± 2.32	*p* = 0.45
*Bgl*-FaNaC									
Left Parietal									
	Dorsal	10.0 ± 8.9	11.5 ± 2.9	15.0 ± 3.0	*p* = 0.61	29.90 ± 2.72	24.07 ± 4.14	14.19 ± 1.45^†,‡^	*p* = 0.002
	Ventral	10.7 ± 6.8	10.7 ± 2.2	15.3 ± 3.8	*p* = 0.60	35.67 ± 4.45	23.07 ± 2.31^†^	15.56 ± 2.19^†,‡^	*p* = 0.006
Visceral									
	Dorsal	12.0 ± 2.6	6.8 ± 1.9	14.5 ± 3.0	*p* = 0.01	24.29 ± 3.39	19.15 ± 2.11	11.21 ± 2.13^†,‡.^	p = 0.005
	Ventral	18.7 ± 9.0	20.3 ± 3.1	17.5 ± 5.2	*p* = 0.90	37.50 ± 5.17	24.89 ± 1.54	15.64 ± 1.95^†,‡^	*p* = 0.011

All tests performed with Brown-Forsythe one-way ANOVA (*df* 2,7). Dunnett’s multiple comparison *post hoc* test. Values shown as $\bar{x}$ ± SEM.

^†^ Significant difference from control values (*post hoc* test *p* < 0.05). ^‡^ Significant difference between infected time points (*post hoc* test *p* < 0.05).

Unlike the heptapeptide precursor, expression of the *Bgl-*FaRP1 tetrapeptide precursor was affected by infection by *S*. *mansoni* (Table 1). The B group of the left parietal ganglion and the E group of the visceral ganglion were analyzed to determine whether these effects occurred generally or in specific cell groups. No changes in the number of cells expressing *Bgl*-FaRP1 or their mean intensity were detected in the left parietal Bgp (Table 1). However, the number of visceral ganglion Egp cells expressing the *Bgl-*FaRP1 tetrapeptide precursor increased at 35 dpi (Fig 13A–13C and 13D; control: 14.7 ± 3.2 cells; 20 dpi: 16.8 ± 2.1 cells; 35 dpi: 22.0 ± 1.7 cells; ANOVA: *F*_(2,7)_ = 7.29; *p* = 0.037). Mean gray values of the Egp *Bgl-*FaRP1 HCR signals were not significantly changed (control: 7.57 ± 2.17; 20 dpi: 6.34 ± 1.68; 35 dpi: 7.01 ± 3.27; ANOVA: *F*_(2,7)_ = 0.29; *p* = 0.45; Fig 13E). Together, these observations indicate that expression of the *Bgl*-FaRP1 tetrapeptide (FMRF-NH_2_) precursor is increased in specific cell clusters relatively late in the infection chronology. This increase appears to occur primarily in Egp cells with low *Bgl*-FaRP1 expression levels prior to infection.

As the *Bgl-*FaNaC was not expressed in discrete clusters, changes were assessed on cell numbers and gray values obtained from the entire visceral and left parietal ganglia surfaces (Table 1 and Figs 14 and 15). No significant changes were observed in the number of *Bgl*-FaNaC expressing cells in either ganglion (Table 1). However, overall gray values were decreased on the ventral surface of the visceral ganglion (control: 37.50 ± 5.17; 20 dpi: 24.89 ± 1.25; shedding: 15.64 ± 1.95; ANOVA: *F*_(2,7)_ = 32.47; *p* = 0.01; Fig 14A–14D). *Bgl-*FaNaC mRNA labeling was also decreased on the dorsal surface of the visceral ganglion of infected snails (control: 24.29 ± 3.39; 20 dpi: 19.15 ± 2.11; shedding: 11.21 ± 2.13; ANOVA: *F*_(2,7)_ = 18.80; *p* = 0.005; Fig 14E–14H). While there was an apparent tendency toward lower expression at 20 dpi on both surfaces, the decreases only reached significant values at 35 dpi.

*Bgl-*FaNaC expression was also decreased in the left parietal ganglion of infected snails (Table 1). On the dorsal surface, decreased overall gray values reached significance at 35 dpi (control: 29.90 ± 2.72; 20 dpi: 24.07 ± 4.14; 35 dpi: 14.19 ± 1.45; ANOVA: *F*_(2,7)_ = 22.50; *p* = 0.002; Fig 15A–15D). *Bgl-*FaNaC expression was also decreased on the ventral surface of the left parietal ganglion (control: 35.67 ± 4.45; 20 dpi: 23.07 ± 2.31; 35 dpi: 15.56 ± 1.31; ANOVA: *F*_(2,7)_ = 33.93; *p* = 0.006; Fig 15E–15K). The decrease in *Bgl-*FaNaC expression on the ventral surface of the left parietal ganglion reached significant levels at 20 dpi (Fig 15H).

## Discussion

### Properties of the *Bgl-*FaNaC

The FaNaC receptors that have been studied to date exhibit at least a 35-fold range of efficacy, with EC_50_ values for FMRF-NH_2_ varying from 2 x 10^−6^ M in *Cornu aspersum* [25] to 7 x 10^−5^ M in *Planorbella trivolvis* [41]. The EC_50_ of 3.3 x 10^−4^ M observed for the *Biomphalaria* FaNaC in the present investigation may be interpreted in the context of structure-activity studies on the *Cornu* and *Planorbella* receptors. Chimeras constructed from these receptors indicated that the peptide recognition site is located in the extracellular region following TM1 (shaded red in Fig 2; [44,45]. Within this region, site-directed mutagenesis was used to substitute *Planorbella* amino acids for the *Cornu aspersum* residues at positions Y131Q, N134T, or I160F (enclosed by rectangles in Fig 2; [46]). Each substitution produced a FMRF-NH_2_ EC_50_ value that was significantly higher than the native *C*. *aspersum* receptor [46]. Notably, these three ‘low affinity’ residues are conserved between the planorbids *Planorbella* and *Biomphalaria*. A fourth substitution that also significantly reduced the affinity of FMRF-NH_2_, D154K (dashed rectangle, Fig 2), is not shared between *Planorbella* and *Biomphalaria* and may contribute to the apparent 4-fold difference in their efficacy.

Divergent peptide recognition sequences could also account for species differences in agonist specificity. FLRF-NH_2_, which is present in two copies on the *B*. *glabrata* FMRF-NH_2_ precursor [35], activates the *C*. *aspersum* FaNaC with an EC_50_ of 11 μM [25]. In contrast, responses of the *Planorbella* FaNaC to FLRF-NH_2_ were negligible [41], in agreement with our observations on *Biomphalaria*. The high level of sequence conservation between the planorbids *Planorbella* and *Biomphalaria* (>90%) may therefore confer an extraordinary degree of agonist specificity in addition to the reduced efficacy of FMRF-NH_2_. The sensitivity of the *Bgl-*FaNaC to amiloride was not tested. Interestingly, while amiloride blocks the FaNaC of *Cornu aspersa* [25], it potentiates responses to FMRF-NH_2_ in the *Planorbella* FaNaC [41]. Clearly, even within the panpulmonates, the FaNaCs exhibit striking divergent properties.

### Localization of the *Bg*FaNaC

Our findings that *Bgl-*FaNaC mRNA was confined to cell bodies and its abundant expression in the subesophageal (visceral and parietal) ganglia were consistent with observations in *Planorbella trivolvis* and *Helix aspersa* [51]. The predominant localization of the *Bgl-*FaNaC protein to neuronal processes was also in agreement with immunohistochemical observations in *Planorbella trivolvis*. When FMRF-NH_2_ was applied to isolated giant dopaminergic neurons (GDN; corresponding to LPeD1 of *Biomphalaria*; Fig 6A and 6B) large inward currents were produced with focal application near the axon hillock, leading to the suggestion that newly synthesized membrane channels were inserted at a high density prior to translocation to distal sites [51].

It is well established that the FMRF-NH_2_ related peptides participate in multiple neural circuits in gastropods, including the control of feeding motor programs [54,55,56], male mating behavior [57,58,59], and cardiorespiratory regulation [60,61]. *Bgl-*FaNaC involvement in each of these circuits was supported by its expression patterns in the buccal (Fig 9), cerebral (Fig 10), and visceral (Fig 11) ganglia, respectively. In the feeding and cardiovascular networks, *Bgl-*FaNaC was expressed in neurons that were in close proximity to cells that express the *Bgl-*FaRP1 precursor.

Co-expression of the *Bgl-*FaNaC and the message for the FMRF-NH_2_ tetrapeptide precursor was rare, but instances were detected in regions of the cerebral ganglion that control male mating behavior (Fig 10). Such co-expression of an agonist and its receptor could enable neurons to form autapses (see [62]). Excitatory autaptic signaling has been shown to produce long-lasting after-discharges in gastropod feeding and reproductive systems [63,64,65]. Autapses are proposed to provide a mechanism whereby a brief stimulus can produce a prolonged response required to drive a motor circuit. In gastropods, male copulation consists of a stereotyped sequence of actions, including preputium eversion, probing, penis eversion, and intromission [53]. Each action lasts for several minutes, probably persisting after termination of its initiating stimulus. Interestingly, application of FMRF-NH_2_ to the water surrounding *Biomphalaria* caused preputium eversion, a behavior that lasts several minutes in the copulatory sequence [66]. The larger cerebral neurons in which *Bgl-*FaNaC and *Bgl-*FaRP1 are co-expressed (Fig 10G–10I) could provide opportunities to examine the involvement of FaNaC receptors in autapses.

### Response to infection

Due to the pleiotropic functions of the FaRPs in gastropods, this neuropeptide signaling system is considered a potential target for schistosome larvae [67,68]. The increase in *Bgl-*FaRP1 expression observed here (Fig 13 and Table 1) agrees with previous studies that measured neuropeptide responses to infection in gastropods. In the host-parasite interaction between the pulmonate snail *Lymnaea stagnalis* and the avian schistosome *Trichobilharzia ocellata*, significant increases in FMRF-NH_2_ gene expression were measured across the post-infection chronology [69]. The early onset of this increase (>300% at five hours) was suggested to reflect a direct effect of parasitism on the host brain. A lower increase at later time points (<100% at 6 and 8 weeks post-infection) was proposed to contribute to the schistosome survival strategy during the shedding stage of infection [69], when host energy resources are redirected toward the large numbers of cercariae inhabiting the snail (see [70,71]).

Elevated levels of FMRF-NH_2_ were also detected with liquid chromatography tandem mass spectrometry (LC-MS/MS) in *B*. *glabrata* nervous systems at 12 days post-infection with *S*. *mansoni* [72]. Of 39 CNS peptides that exhibited >1.5-fold changes, FMRF-NH_2_ was one of only 6 that was increased. It was proposed that the increased expression of FMRF-NH_2_ could contribute to enhanced metabolic activity during the pre-patent phase of infection [72].

Our observations suggest one potential source of elevated levels of FMRF-NH_2_ in infected snails (Fig 15l). Increased precursor expression was limited to *Bgl-*FaRP1 (Fig 13 and Table 1), the tetrapeptide precursor that encodes FMRF-NH_2_, the sole *Bgl-*FaNaC agonist [35]. No changes in expression were observed for the heptapeptide precursor *Bgl-*FaRP2 (Fig 12 and Table 1). Moreover, the increased expression was limited to a subset of *Bgl-*FaRP1expressing neurons in the visceral ganglion and was primarily observed late in the infection chronology. In contrast, down-regulation of the *Bgl-*FaNaC receptor appeared to commence earlier and occurred throughout the visceral and left parietal ganglia.

We propose that increased *Bgl-*FaRP1 expression could reflect a compensatory mechanism that occurs in response to decreased receptor expression (Fig 15l). Such homeostatic increases in neuropeptide expression would only occur in neurons that are presynaptic to neurons that express *Bgl-*FaNaC. Diverse mechanisms, including altered neurotransmitter release, are known to contribute to maintenance of signals following perturbation of synapses (see [73,74]). In the case of rapid signaling by FMRF-NH2 via the *Bgl-*FaNaC, such compensatory mechanisms could include increased precursor gene expression in response to decreased availability of postsynaptic receptors (Fig 15l). Future studies should explore the role of *Bgl-*FaNaC in synaptic signaling and examine whether such signaling is maintained despite reduced receptor expression levels following infection. The participation of this signaling pathway in multiple vital physiological and behavioral circuits, coupled with its extraordinary agonist specificity and apparent limitation to heterobranch taxa, could lead to novel strategies for control of snail pests.
